# Baicalin, the major component of traditional Chinese medicine Scutellaria baicalensis induces colon cancer cell apoptosis through inhibition of oncomiRNAs

**DOI:** 10.1038/s41598-018-32734-2

**Published:** 2018-09-27

**Authors:** Yili Tao, Shoubin Zhan, Yanbo Wang, Geyu Zhou, Hongwei Liang, Xi Chen, Hong Shen

**Affiliations:** 1 Nanjing University of Chinese Medicine, 282 HanZhong Road, Nanjing, Jiangsu 210046 China; 2 Department of Gastroenterology, Affiliated Hospital of Nanjing University of Chinese Medicine, 155 HanZhong Road, Nanjing, Jiangsu 210000 China; 3 School of Life Sciences, Nanjing University, 163 XianLin Road, Nanjing, Jiangsu 210093 China

## Abstract

Colorectal cancer (CRC) is among the most frequently occurring cancers worldwide. Baicalin is isolated from the roots of Scutellaria baicalensis and is its dominant flavonoid. Anticancer activity of baicalin has been evaluated in different types of cancers, especially in CRC. However, the molecular mechanisms underlying the contribution of baicalin to the treatment of CRC are still unknown. Here, we confirmed that baicalin can effectively induce and enhance apoptosis in HT-29 cells in a dose-dependent manner and suppress tumour growth in xenografted nude mice. We further performed a miRNA microarray analysis of baicalin-treated and untreated HT-29 cells. The results showed that a large number of oncomiRs, including miR-10a, miR-23a, miR-30c, miR-31, miR-151a and miR-205, were significantly suppressed in baicalin-treated HT-29 cells. Furthermore, our *in vitro* and *in vivo* studies showed that baicalin suppressed oncomiRs by reducing the expression of c-Myc. Taken together, our study shows a novel mechanism for anti-cancer action of baicalin, that it induces apoptosis in colon cancer cells and suppresses tumour growth by reducing the expression of c-Myc and oncomiRs.

## Introduction

Colorectal cancer (CRC) is one of the most common cancers worldwide^1^. In the United States, it was estimated that there were 132,700 newly diagnosed CRC cases as well as 49,700 CRC-related deaths in 2015^2^, which underscores the need to develop more efficient or complementary treatment^3,4^. Herbal medication is an approach that is gaining big attention for CRC treatment nowadays^2,5^, while botanicals are known to be an important resource for several efficacious chemotherapy agents^6,7^. Thus, identifying non-toxic natural ingredients from herbs is a crucial step in promoting CRC therapeutics^8,9^.

Natural products have recently received attention for the discovery of novel anticancer therapeutic agents as they have long been used as alternative remedies for a variety of diseases, including cancer, with relatively fewer side effects^10,11^. Therefore, identifying natural ingredients to advance anticancer treatment is in prospect. Baicalin (5, 6-dihydroxy-7-O-glucuronide flavone) is a predominant flavonoid isolated from the roots of Scutellaria baicalensis Georgi (Huang Qin) with a defined chemical constitution^12,13^ and various pharmacological activities, including anti-oxidative, anti-viral, anti-inflammatory, anti-HIV and anti-proliferative activities^14–18^. It also has beneficial effects in the treatment of several cancers, including CRC^5^. However, the molecular mechanisms underlying the contribution of baicalin to CRC treatment remain elusive.

MicroRNAs (miRNAs) are a class of 18–22 nucleotides small non-coding RNA molecules that play pivotal roles in development, differentiation, apoptosis, senescence and cell proliferation through post-transcriptional regulation of gene expression^19^. Aberrant expression of miRNAs is known to be associated with a variety of human diseases, such as cardiac disorders, immune-related disorders, neurodegenerative diseases and cancers^20,21^, including CRC^22^. Many oncogenic miRNAs (oncomiRs) that mediate cell growth and tumour progression, including miR-21, miR-23a, miR-17–5p, miR-15b, miR-181b, miR-191 and miR-200c, are upregulated in CRC^23–26^, while others, such as miR-204, miR-34a and miR-126, are found to be downregulated and may function as tumour suppressors^27–29^. The deregulation of various miRNAs is related to tumour diagnosis and prognosis, illustrating that they might provide important references for clinical applications^30–32^.

In the present study, we attempt to demonstrate whether and how baicalin contributes to CRC management. We first confirmed that baicalin effectively enhances apoptosis in HT-29 cells in a dose and time-dependent manner and suppresses tumour growth in xenografted nude mice. Using a miRNA microarray analysis, we further showed that the enhancement of apoptosis is coupled with downregulation of a large number of oncomiRs, including miR-10a, miR-23a, miR-30c, miR-31, miR-151a and miR-205, after baicalin treatment. Finally, we demonstrated the role of c-Myc, which is also suppressed after baicalin treatment, in regulating these oncomiRs both *in vitro* and *in vivo*.

## Results

### Baicalin inhibits cell growth and induces apoptosis in HT-29 colon cancer cells

**Figure 1:**
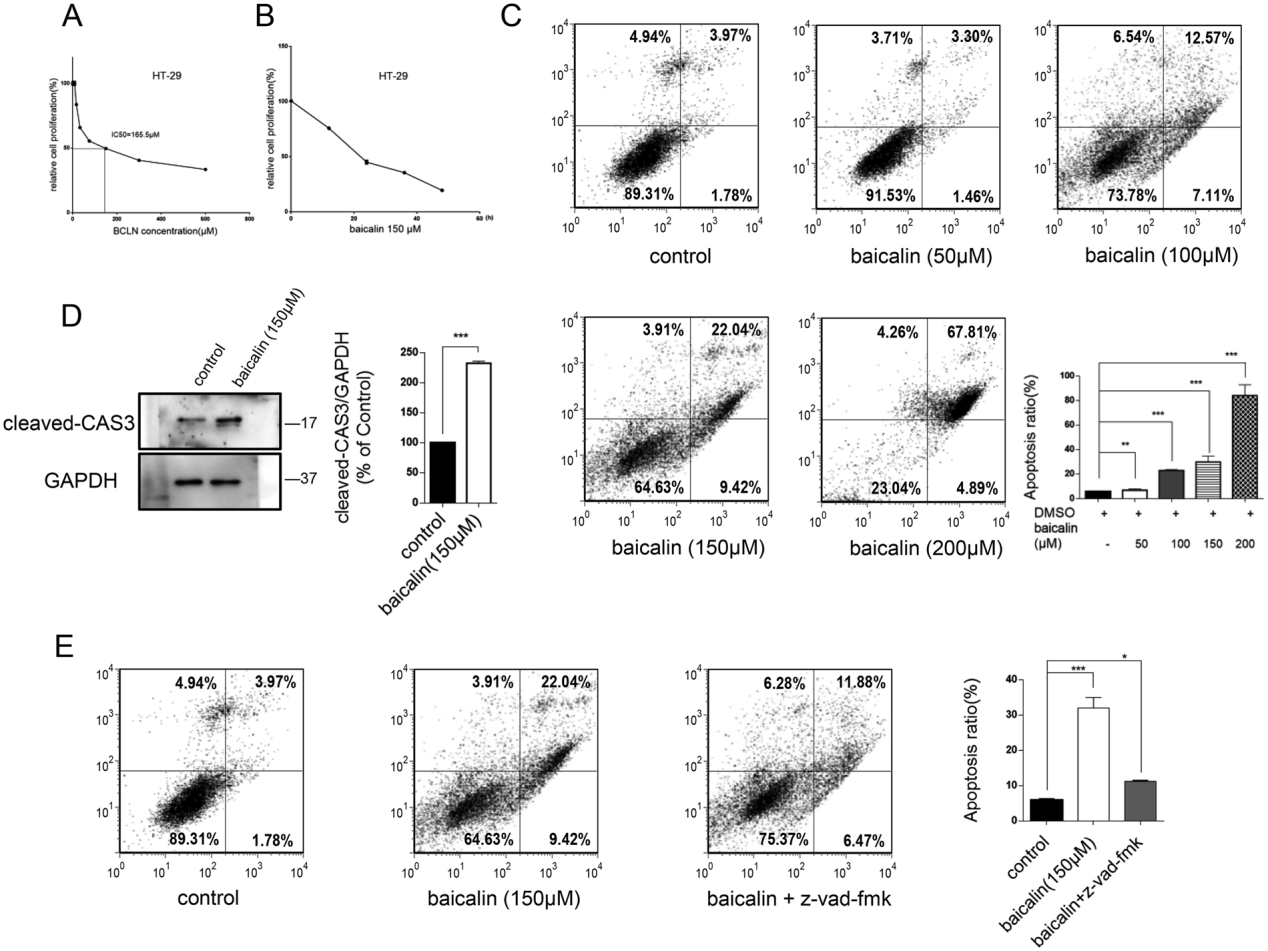
Effects of Baicalin at different dosages on apoptotic induction in HT-29 cells. (**A**) IC 50 of baicalin in HT-29 cells. Cells were treated with various concentrations of baicalin (0–600 µM) and cell viability tests were analyzed by the standard cell counting kit-8 (CCK-8) assay method. (**B**) Cell viability of HT-29 cells treated with 150 µM baicalin for 0, 12, 24, 36 and 48 h was measured by CCK-8 assay. **(C**) Flow cytometric analysis of baicalin-induced apoptosis in HT-29 cells and percentage of apoptotic cells. Cells were cultured overnight in 6-well plates and treated in triplicate with baicalin (50, 100, 150 or 200 µM) for 48 h. (**D**) Cleaved-caspase3 gene expression in baicalin (150 µM) treated HT-29 cells. GAPDH was employed as a loading control. **(E**) Flow cytometric analysis of baicalin-induced and Z-VAD-FMK applied apoptosis in HT-29 cells and percentage of apoptotic cells. *P < 0.05; **P < 0.01; ***P < 0.001.

To investigate the effect of baicalin on human colon cancer cells growth, the cytotoxic efficacy of baicalin was examined *in vitro* using HT-29 cell lines. As is shown in Fig. 1A, baicalin has significant inhibition on growth in HT-29 cells with half-maximal inhibitory constants (IC50) of 165.5 µM, and a time-dependent loss of cell viability after exposure to baicalin was observed (Fig. 1B). To explore whether baicalin inhibits cell viability through the induction of apoptosis, we examined the effect of baicalin on apoptosis of HT-29 cells. We treated HT-29 cells with different concentrations of baicalin (0, 50, 100, 150 and 200 µM) for 24 h and examined the proportion of apoptotic cells via flow cytometry assays. The results revealed that baicalin induced the apoptosis of HT-29 cells in a dose-dependent manner (Fig. 1C). It also induced apoptosis in colon cancer cell lines SW-480 and CACO2 (Supplementary Fig. S2A and B).

Furthermore, we measured cleaved-Caspase3, an apoptotic marker, in baicalin-treated HT-29 cells and found that cleaved-Caspase3 was significantly increased in baicalin-treated HT-29 cells compared with control cells (Fig. 1D). To verify whether apoptosis plays an essential role on cell viability inhibition, apoptotic inhibitor Z-VAD-FMK was applied, and it significantly recovered the viability inhibition induced by baicalin (Fig. 1E). These results demonstrate that baicalin inhibits cell viability through enhancing apoptosis in HT-29 colon cancer cell.

### Baicalin suppresses the expression of a large number of oncomiRs in colon cancer cells

**Figure 2:**
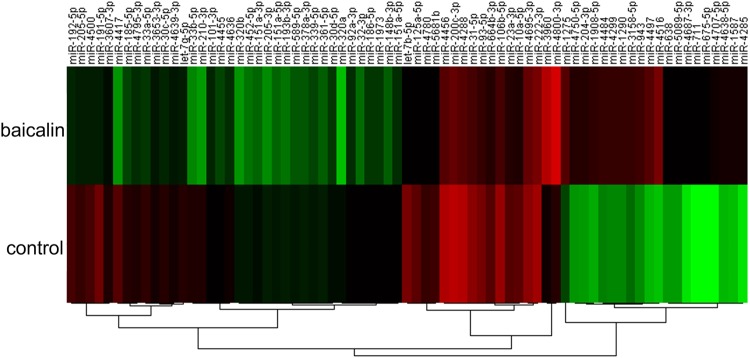
Profile of miRNA expression in Baicalin-treated and untreated HT-29 cells using miRNA microarray technology. The expression of miRNAs is hierarchically clustered on the y-axis, and baicalin treated or control (untreated) HT-29 cells are hierarchically clustered on the x-axis. The relative miRNA expression is depicted by the colour scale. Red indicates upregulation; green indicates downregulation.

Studies have shown that miRNAs play pivotal roles in biological processes, including apoptosis^33^. To verify whether baicalin induces apoptosis through regulating certain miRNAs, we profiled the miRNA expression in a subset of HT-29 cells treated with baicalin using an Exiqon miRCURY LNA microRNA Array. The level of miRNAs differed significantly between control samples and baicalin-treated samples (Fig. 2, Supplementary Table S1). Of the 2000 miRNAs detected on the microarray, 37 miRNAs were found to be significantly downregulated, while only 8 miRNAs were upregulated after baicalin treatment (fold change >2) (Supplementary Table S2). Among the downregulated miRNAs, at least 20 miRNAs are reported as oncomiRs, including miR-92a, miR-222, miR-664b, miR-23a, miR-10a, let-7g, miR-93, miR-192, miR-205, let-7b, miR-191, miR-210, miR-31, miR-30c, miR-205, miR-151a, miR-106b, miR-200c, miR-34a and miR-32^34–45^. Among the upregulated miRNAs, only two miRNAs (miR-204 and miR-638) are reported to have an anticancer effect. Therefore, we focused our study on the downregulated miRNAs.

To obtain a better understanding of the biological functions of differentially expressed miRNAs and their target genes, GO analysis was performed using the database for annotation, visualization and integrated discovery. The genes targeted by differentially expressed miRNAs are strongly enriched for many apoptosis-related processes (FDR <0.05, Supplementary Fig. S1C and E), including regulation of programmed cell death (GO:0043067), regulation of apoptosis (GO:0042981), and positive regulation of apoptosis (GO:0043065) (Supplementary Fig. S1A). Pathway analysis showed similar enrichment of targeted genes for apoptosis-related pathways (Supplementary Fig. S1B, D,F), such as the TGF beta signalling pathway (hsa04350), p53 signalling pathway (hsa04115) and apoptosis (hsa04210). Taken together, these results suggest that baicalin primarily acts to suppress oncomiRs whose targets are putatively involved in regulation of apoptosis.

### miRNAs mimics the pro-apoptotic effects of Baicalin in colon cancer cells

**Figure 3:**
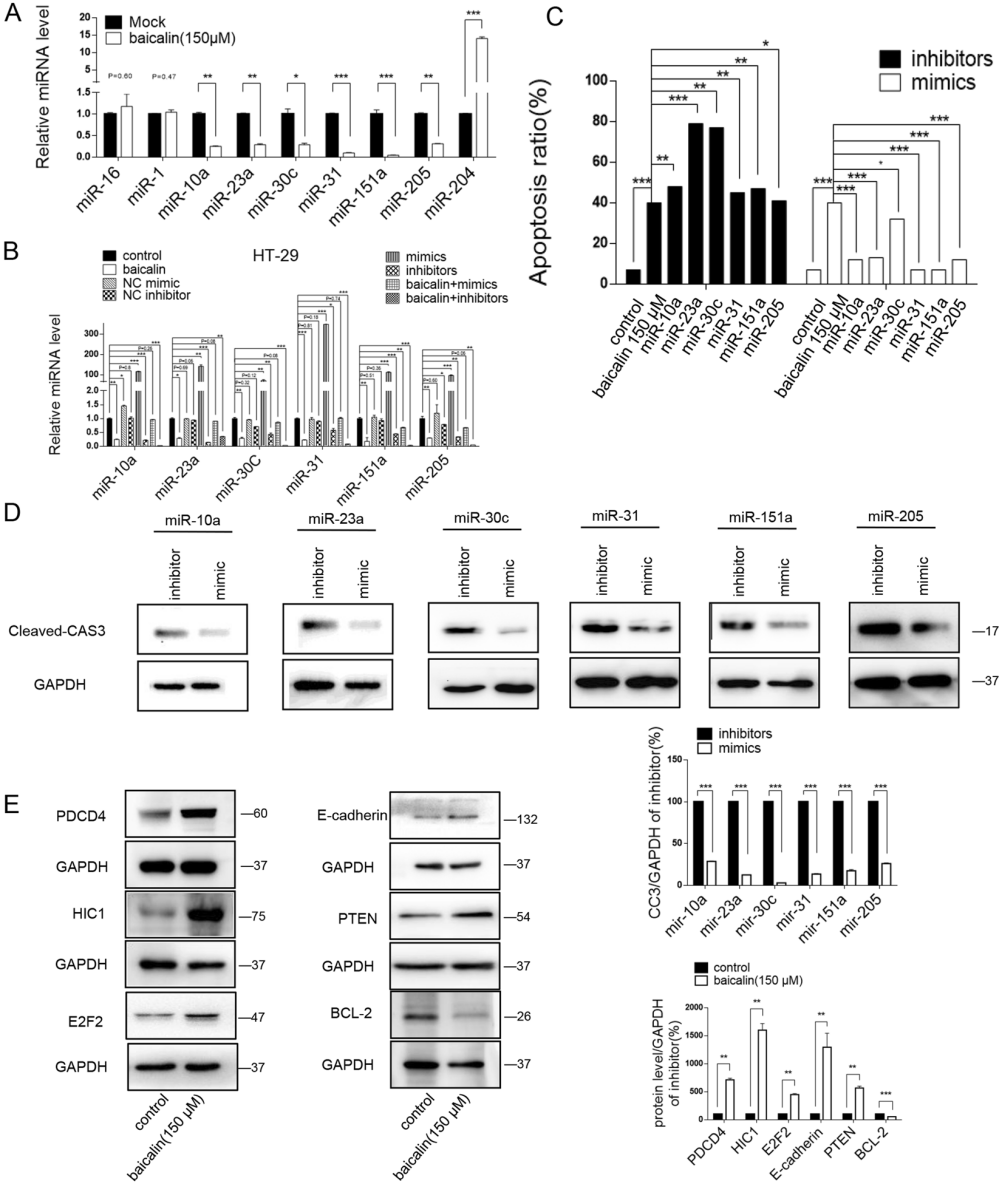
Baicalin suppresses the expression of a large number of oncomiRs in colon cancer cells. (**A**) The relative change in expression levels of representative miRNAs in the baicalin-treated HT-29 cells compared with controls. **(B)** qRT-PCR analysis of downregulated miRNA expression in baicalin treated or untreated HT-29 cells that were transfected with their miRNA-mimics, miRNA-inhibitors, mimic NCs, and inhibitor NCs. The miRNA expression in uninduced NC transfected cell was normalized as 1. **(C)** Flow cytometric analysis of the transfected cells and percentage of apoptotic cells. **(D)** Western blot analysis of cleaved-Caspase3 protein expression in the transfected cells and quantitative analysis. **(E)** Western blot analysis of PDCD4, HIC1 and BCL-2 in baicalin-treated HT-29 cells and quantitative analysis. *P < 0.05, **P < 0.01, ***P < 0.001.

Next, the miRNA Array result was validated by TaqMan probe-based qRT-PCR analysis. Levels of miR-10a, miR-23a, miR-30c, miR-31, miR-151a and miR-205 in baicalin treated HT-29 cells were remarkably decreased and miR-204 was increased in baicalin-treated cells compared with the control cells, while the expression of two control miRNAs (miR-1 and miR-16) had no significant changes between baicalin-treated cells and control cells (Fig. 3A). To test the robustness of the effect, two additional colon cancer cell lines (SW-480 and CACO2) were used to repeat the above experiments and consistent results were observed (Supplementary Fig. S2C and D).

To investigate whether baicalin induced cell apoptosis through altering the expression of these miRNAs, we transfected HT-29 cells with miRNA mimics or miRNA inhibitors of miR-10a, miR-23a, miR-30c, miR-31, miR-151a or miR-205, individually, along with baicalin. First, compared with the baicalin only group, the cellular levels of miR-10a, miR-23a, miR-30c, miR-31, miR-151a and miR-205 were dramatically increased in HT-29 cells after transfection with corresponding mimics and dropped significantly after treatment with corresponding antisense (Fig. 3B). To test the robustness of the effect, two additional colon cancer cell lines (SW-480 and CACO2) were used to repeat the above experiments and consistent results were observed (Supplementary Fig. S2E and F).

As anticipated, transfection of miR-10a, miR-23a, miR-30c, miR-31, miR-151a and miR-205 inhibitors separately could significantly induce apoptosis in baicalin treated HT-29 cells, whereas transfection of their mimics markedly attenuated baicalin induced apoptosis (Fig. 3C, Supplementary Fig. S2G). In line with this, we also checked the level of apoptotic-related cleaved-Caspase3 and found it consistently upregulated in the miRNA inhibitors-transfected cells compared with the miRNA-mimics treated cells (Fig. 3D). To further confirm the effect of the above ectopic expression miRNAs on baicalin-induced apoptosis in colon cancer cells, some apoptosis related target genes of these miRNAs were checked, such as HIC1 (miR-23a^46^), PDCD4 (miR-23a and miR-205^47^), PTEN (miR-10a^48^), E2F2 (miR-31^49^), E-cadherin (miR-151a^50^) and BCL-2 (miR-204^51^). As expected, the level of HIC1, PDCD4, PTEN, E2F2 and E-cadherin were significantly upregulated, while BCL-2 was downregulated in baicalin-treated HT-29 cells compare to untreated control cells (Fig. 3E). Taken together, these results demonstrate that baicalin promotes the apoptosis of colon cancer cells through a miRNA-dependent manner.

### Baicalin suppresses c-Myc expression to downregulate oncomiRs

**Figure 4:**
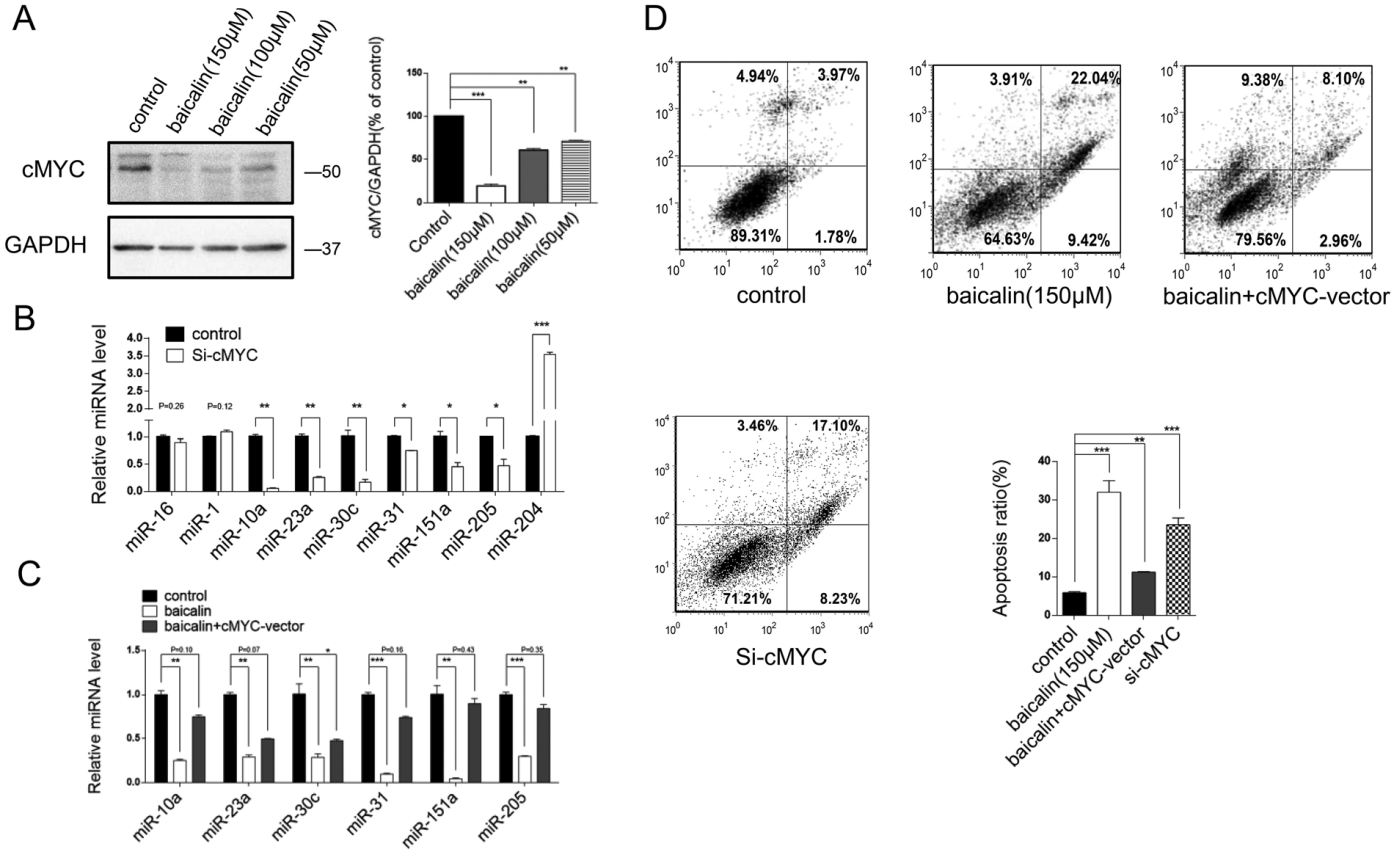
Baicalin represses c-Myc expression to downregulate oncomiRs. (**A**) Western blot of c-Myc expression in baicalin-treated HT-29 cells: representative image and quantitative analysis. (**B**) The relative change in expression levels of representative miRNAs in the c-Myc siRNA-transfected HT-29 cells compared with controls. (**C**) The relative change in expression levels of representative miRNAs in HT-29 cells co-treatment with baicalin and c-Myc overexpression vector compared with controls. **(D)** Flow cytometric analysis of the baicalin treated, c-Myc-vector transfected and c-Myc siRNA transfected HT-29 cells and percentage of apoptotic cells. *P < 0.05; **P < 0.01; ***P < 0.001.

Subsequently, we investigated the underlying mechanism accounting for the observed global inhibition of oncomiRs induced by baicalin. Recent studies have reported that the oncogenic transcription factor c-Myc induces oncomiR expression and contributes to tumourigenesis^52^. C-Myc was suppressed in a dose-dependent manner in HT-29 cells treated with baicalin at various concentrations (0, 50, 100 and 150 µM) (Fig. 4A). It was also confirmed in colon cancer cell lines SW-480 and CACO2 (Supplementary Fig. S3A and B). We further verified the consequence of c-Myc downregulation on the level of these miRNAs by directly inhibiting c-Myc expression. Cells were transfected with different concentrations of c-Myc siRNAs to mimic baicalin treatment (Supplementary Fig. S3C). Consistent with baicalin treatment, the expression levels of miR-10a, miR-23a, miR-30c, miR-31, miR-151a and miR-205 were significantly decreased along with c-Myc, while the expression levels of miR-16 and miR-1 did not significantly change, and miR-204 was significantly increased in c-Myc siRNA-treated cells compared with the untreated control cells (Fig. 4B). To further validate the correlation between oncomiR expression and c-Myc expression, we assessed the miRNA levels in HT-29 cells after co-treatment with baicalin and c-Myc overexpression vector. As expected, overexpression of c-Myc rescued the reduction of these miRNAs induced by baicalin (Fig. 4C). As a consequence, c-Myc siRNA induced cell apoptosis as well as baicalin did, while c-Myc overexpression attenuated the inhibitory effect of baicalin on cell apoptosis (Fig. 4D). These results suggest that baicalin represses c-Myc expression to inhibit the expression of oncomiRs in colon cancer cells.

### Baicalin suppresses tumour growth in xenografted nude mice

**Figure 5:**
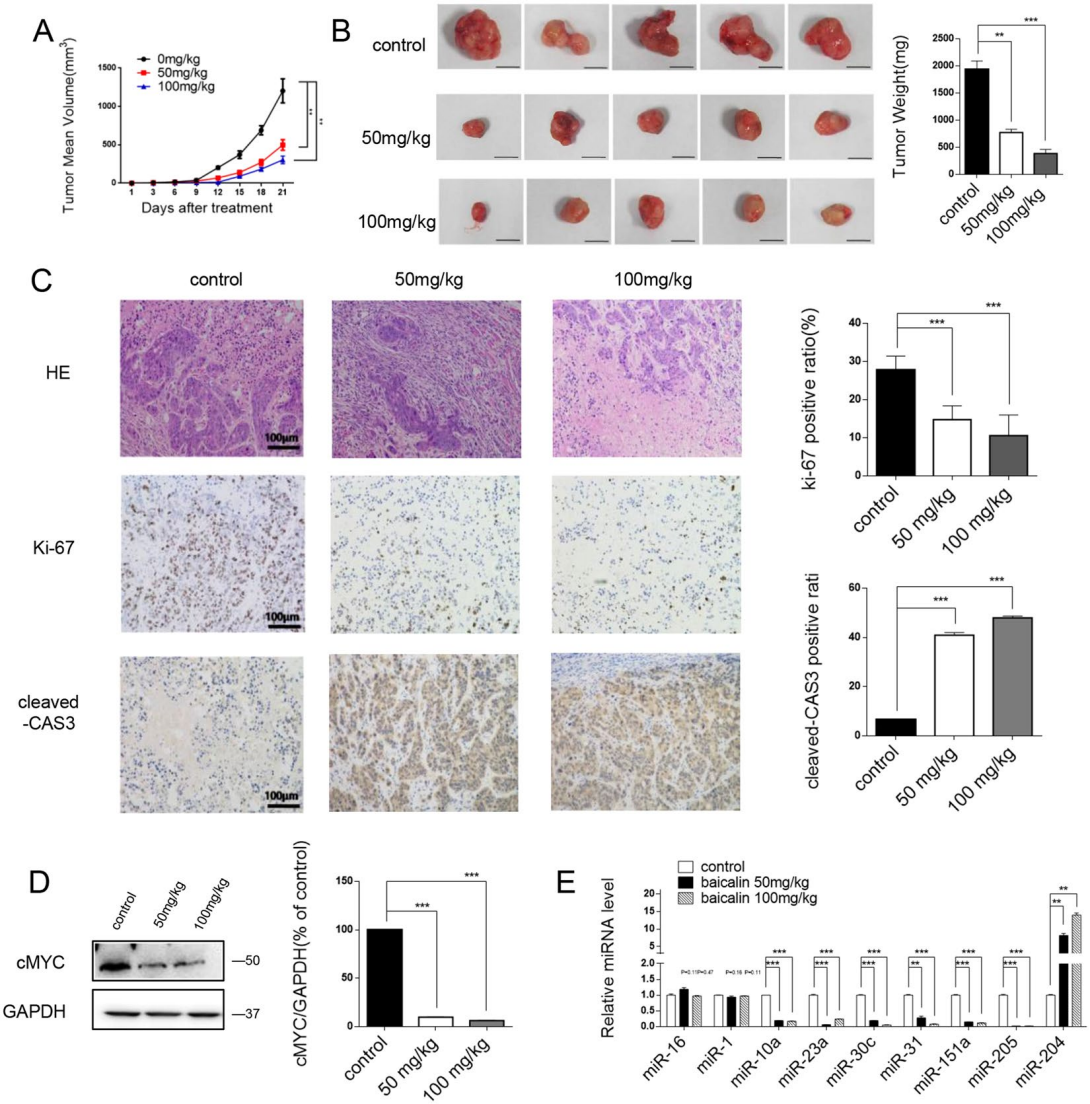
*In vivo* evidence for the anti-tumour effect of baicalin in nude mice xenograft model. HT-29 cells (2 × 10^6^ cells per 0.1 mL) were implanted subcutaneously into 6-week-old xenograft mice. After inoculation for one week, the mice were randomly treated with vehicle (10% DMSO and 90% PBS) or baicalin at 50 mg/kg and 100 mg/kg daily intraperitoneally (5 mice per group). **(A**) The time course of tumor growth in implanted mice. Tumor volume was measured every 3 days for 21 days after baicalin treatment. (**B**) Representative images of the tumours from the implanted mice and quantitative analysis of the tumour weights. **(C)** H&E-stained sections and immunohistochemical staining for Ki-67 and cleaved-caspase3 in the tumours from implanted mice and quantitative analysis. **(D)** Western blotting analysis of c-Myc protein levels in the tumours from implanted mice and representative image. **(E**) Quantitative RT-PCR analysis of representative miRNAs in the tumours from implanted mice. *P < 0.05; **P < 0.01; ***P < 0.001.

The *in vivo* efficacy of baicalin against tumour growth was further investigated to confirm the above-mentioned results. Xenograft tumour models in which HT-29 cells were injected into nude mice were constructed, and a week later mice were treated with various concentrations of baicalin (*i.p*., 50 and 100 mg/kg) daily for 21 days. Compared with the control group, baicalin had a significant inhibitory effect on tumour growth (Fig. 5A). After 21 days of treatment, all the nude mice were sacrificed, and xenografts were removed and weighed. Compared with the control group, the mean tumour weight was significantly lighter in baicalin-treated mice (Fig. 5B), suggesting that baicalin can suppress the growth of xenografted colon tumours in nude mice. Furthermore, haematoxylin and eosin (H&E) staining of xenograft tissues showed fewer mitotic cells in the group treated with baicalin compared with the control group, along with a smaller number of inflammatory cells and less necrocytosis (Fig. 5C). The cell proliferation rate evaluated by immunocytochemistry with the mouse monoclonal antibody Ki-67 revealed that the percentage of Ki-67-positive tumour cells was significantly declined in the baicalin treated group (Fig. 5C). The percentage of cleaved-Caspase3 in the sectioned tumor tissues was measured to determine the effect of baicalin on apoptosis. Considerably stronger cleaved-Caspase3 staining intensities were detected in baicalin-treated tumor tissues relative to control tissues (Fig. 5C). To determine whether the reduced tumour growth rate following baicalin treatment could be explained by the repression of c-Myc, we examined the expression level of c-Myc in tumour sections with western blot, and the expression levels of c-Myc were significantly decreased (Fig. 5D). Consistent with the *in vitro* findings, miR-10a, miR-23a, miR-30c, miR-31, miR-151a and miR-205 were significantly downregulated in baicalin-treated mice, while miR-16 and miR-1 exhibited no significant change, and miR-204 was significantly upregulated (Fig. 5E). These results confirm that baicalin inhibits tumour growth by repressing the expression of c-Myc and oncomiRs to induce apoptosis.

## Discussion

CRC is the second most prevalent cancer across the world, and it’s also the second leading cause among all the cancer related death in the United States^1,2^. Surgery, radiation and chemotherapy are conventional treatments of CRC management^53^. With high incidents and complex characteristics, complementary therapies such as herbal medication are in desperate need^54^. Natural products have been a rich source of valuable medical agents. Over 50% of the currently efficient drugs ^55^or compounds are related to natural products, and in the case of cancer this proportion surpasses 60%^11^. Herbal medicine has substantial contributed to CRC treatments, and more novel ingredients with promising anticancer activities are likely to be found in plant sources.

Scutellaria baicalensis is one of the most widely used medicinal plants in traditional Chinese medicine^56^. The representative components of  S. baicalensis are a group of flavonoids including glycosides (baicalin, wogonoside) and their aglycon metabolites (baicalein and wogonin)^12,13^. The anticancer activities of S. baicalensis and its chemical components have been reported^5,57–59^. In this study, we investigated the molecular mechanisms underlying the contribution of baicalin to CRC treatment. In our pilot study, we found that baicalin enhances apoptosis in HT-29 colon cancer cells in a dose-dependent manner.

Dysregulated expression of c-Myc contributes to the genesis of a large fraction of human tumours^60^. Its deregulation, overexpression or misexpression generally promotes cellular proliferation and growth, and inhibits cell differentiation^61^. Dysregulation of several miRNAs has also been reported to be controlled by c-Myc during carcinogenesis^33,62^. Through the Exiqon miRCURY LNA microRNA Array, we found that baicalin could suppress a large number of oncomiRs, while functional analysis of the target genes of these miRNAs showed that these genes were significantly enriched in apoptosis. Our data also confirmed that baicalin reduced the expression of c-Myc in colon cancer cells, which resulted in the downregulation of the expression of oncomiRs^63–70^. Studies have shown that miR-10a is correlated with aggressive progression and poor prognosis in cervical cancer^67^ and is overexpressed in medullary thyroid carcinomas^68^. MiR-23a is found to be upregulated in many types of cancer. It is a significant oncomiR that induces proliferation, migration and represses apoptosis^71,72^ through inhibiting metastasis suppressor gene expression. Furthermore, an advanced clinical stage, the depth of invasion, and lymph node metastasis is closely related to the upregulation of miR-23a expression, which indicates that it could be a biomarker for the prognosis of CRC^73^. Mir-30c is reported to promote the invasive phenotype of metastatic breast cancer cells^74^. MiR-31 is frequently altered in numerous cancers and plays an oncogenic function^64,65^. It is also significantly upregulated in colon cancer cell lines, and its expression level is correlated with the stage of CRC tumours^75^. As an oncomiR, miR-205 may promote the clinical progression of different cancers, including CRC^76,77^. Thus, it is reasonable to observe the suppression of miR-10a, miR-23a, miR-30c, miR-31, miR-151a and miR-205 after baicalin treatment, accompanied by enhanced apoptosis. Baicalin also upregulates miR-204 expression significantly. Downregulation of miR-204 is found in many solid tumours, namely bladder cancer, primary melanomas, non-small cell lung cancer, gastric cancer, glioma, head and neck cancer and endometrioid endometrial cancer^27,78,79^. Studies have also revealed that miR-204 primarily acts as a tumour suppressor by inducing apoptosis to inhibit tumour initiation, progression and drug resistance. Our *in vivo* data also confirmed that baicalin inhibits tumour growth by repressing c-Myc, which in turn resulted in the decline of oncomiRs in the xenograft model.

In conclusion, our studies confirm that baicalin induces apoptosis in colon cancer cells by inhibiting c-Myc expression and simultaneously downregulating the expression of many apoptosis-related oncomiRs. These findings identify a novel relationship between baicalin, c-Myc and miRNA, which provides a reasonable and clear explanation for the contribution of baicalin in the treatment of colon cancer, and it may also provide a new thought for CRC treatment and anticarcinogen development.

## Materials and Methods

### Cell culture

The human colon cancer cell line HT-29 obtained from the Shanghai Institute of Cell Biology, Chinese Academy of Sciences (Shanghai, China) were maintained in RPMI 1640 with 10% foetal bovine serum (GIBCO, CA, USA) and incubated in 5% CO_2_ at 37 °C in a water-saturated atmosphere.

### Cell Viability Assay

The cell viability tests were analyzed by the standard cell counting kit-8 (CCK-8) assay method. HT-29 cells were seeded into a 96-well plate (1 × 10^4^ cells per well) in cell culture medium. After 12 h, the medium was replaced with 100 µL of fresh medium containing different concentrations of baicalin (0–600 µM) and incubated for a further 24 h, or incubated with baicalin at 150 µM for 0–48 h. Cells were then washed twice with PBS and incubated with 110 µL fresh medium containing 10 µL CCK-8 solutions for a further 3 h. Finally, the medium was removed and the absorbance at 460 nm was measured using a microplate reader (TECAN M200 infinite Pro). Note that all experiments were conducted in triplicate, and error bars shown represent the standard error of independent experiments. The cell viability (%) was calculated by the following formula, where [A] is the average absorbance: Cell Viability (%) = ([A]_460(sample)_ − [A]_460(blank)_)/([A]_460(control)_ − [A]_460(blank)_)*100.

### Cell Transfection

To achieve miR-10a, miR-23a, miR-30c, miR-31, miR-151a and miR-205 overexpression, cells were transfected with miRNA mimics, while knockdown of these miRNAs was achieved by transfecting cells with miRNA antisense. Synthetic miRNA mimic, antisense and scrambled negative control RNAs (pre-miR-control and anti-miR-control) were purchased from GenePharma (Shanghai, China).

The siRNA sequences targeting human c-Myc cDNA were designed and synthesized at RiboBio (Guangzhou, China). The siRNA sequences are listed below: siRNA587, 5′-CGTCCTCGGATTCTCTGCTC-3′;siRNA630,5′-TACAACACCCGAGCAAGGAC-3′;siRNA1094,5′-CGGGAAAAAGAACGGAGGGA-3′; and siRNA1624,5′-GGACTTGTTGCGGAAACGAC-3′. A scrambled siRNA was included as a negative control. The cells were seeded in 6-well plates and transfected using Lipofectamine 3000 (Invitrogen) according to the manufacturer’s instructions.

### Baicalin treatment

Baicalin (Sigma) was dissolved in dimethyl sulfoxide (DMSO) in a 100 µM stock solution, stored at −20 °C, and diluted to different concentrations with culture medium right before experimental use. A same volume of DMSO with a final concentration of 0.08% was added to the controls.

### Flow cytometry analysis of apoptosis

HT-29 cells were treated with baicalin at different concentrations (0, 50, 100,150 and 200 µM) or c-Myc siRNA or c-Myc vectors for 48 h. Pan-caspase inhibitor Z-VAD-FMK (Biovision, USA) was applied following manufacturer’s procedure. Cells were harvested and washed twice with PBS, and then re-suspended in binding buffer followed by staining with Annexin V-FITC/PI at room temperature for 15 min in the dark (BD Biosciences, CA, USA). Apoptotic cells were evaluated afterwards by gating PI- and Annexin V-positive cells on a fluorescence-activated cell-sorting (FACS) flow cytometer (BD Biosciences, CA, USA), and total apoptotic cells were counted as the sum of early apoptotic (PI− AV+) and late apoptotic (PI+ AV+) cells. All experiments were performed in triplicate.

### RNA isolation and qRT-PCR

Total RNA was extracted from cells and tissues with TRIzol (Invitrogen, Carlsbad, CA). RNA was reverse-transcribed to cDNA with AMV reverse transcriptase (TaKaRa, Dalian, China) and a stem-loop RT primer (Applied Biosystems). TaqMan miRNA probes (Applied Biosystems, Foster City, CA) were used to quantify miRNAs. Real-time PCR was performed using a TaqMan PCR kit on an Applied Biosystems Roche Sequence Detection System. Relative expression of miRNA relative to the internal control U6 was determined using the 2^−ΔΔCT^ method: ΔΔC_T_ = (C_TmiRNA_ − C_TU6_)_target_ − (C_TmiRNA_ − C_TU6_)_control_. All reactions were performed in triplicate.

### Protein isolation and western blotting

All cells were washed with PBS, tissue samples were frozen solid with liquid nitrogen, and were subsequently grounded into powder. Protein extraction was achieved using RIPA Lysis buffer (Beyotime, China) supplemented with a Protease and Phosphatase Inhibitor Cocktail (Thermo Scientific 78440). Extracted protein concentration was calculated using a Pierce BCA protein assay kit (Thermo Scientific, Rockford, IL, USA). The protein levels were analysed via western blotting using the corresponding antibodies, and were normalized to GAPDH expression. Antibodies were purchased from the sources below: anti-c-Myc (9E10) (sc-40, Santa Cruz, CA, USA), anti-cleaved-Caspase3 (9664, Cell Signaling Technology), anti-PDCD4 (9535, Cell Signaling Technology), anti-HIC1 (sc-271499, Santa Cruz), anti-E-cadherin (sc-8426, Santa Cruz), anti- E2F2 (ab 138515, Abcam), PTEN (138G6, Cell Signaling Technology), anti-BCL-2 (sc-7382, Santa Cruz) and anti-GAPDH (sc-365062, Santa Cruz). The protein bands were analysed using Image-Pro Plus software.

### Profiling of miRNA expression

MiRCURY LNA Array system was used to detect the expression profiles of miRNAs in HT-29 cells treated with 150 µM baicalin (version. 18.0, Exiqon Inc., Woburn, MA, USA), conducted by KangChen Bio-tech, Inc. (Shanghai, China). RNA samples were labelled using a miRCURY Hy3/Hy5 Power labelling kit (Exiqon Inc., Woburn, MA, USA) and hybridized on the miRCURY LNA Array station. Axon GenePix 4000B microarray scanner (Molecular Devices, LLC, Sunnyvale, CA, USA) and GenePix Pro version 6.0 was used to scan and read the raw data. Signal intensity was calculated after background subtraction, and median intensity was applied to summarize the replicated spots in the same image. The median normalization method was used to obtain normalized data (foreground minus background divided by median intensity in one library). The threshold value for significance, with a fold change >2 and FDR <0.05, was used to define miRNA upregulation or downregulation. GO and KEGG pathway enrichment analysis was performed with DAVID bioinformatics tools.

### Establishment of tumour xenografts in mice

Mice were purchased from the Model Animal Research Center of Nanjing University (Nanjing, China), and were maintained under specific pathogen-free conditions in accordance with institutional policies at Nanjing University. All the animal procedures were approved by the Animal Experimentation Ethics Committee of Nanjing University. HT-29 cells were injected subcutaneously into six-week-old male nude mice (2 × 10^6^ cells per mouse) to induce tumor formation. After inoculation for one week, the mice were treated with baicalin (50 mg/kg or 100 mg/kg) or vehicle (10% DMSO and 90% PBS) by intraperitoneally injection once every day for 3 weeks, with 5 mice in each group. Tumor sizes were measured every 3 days, and the ellipsoid volume was calculated as follows: Volume = (length) × (width) × (width)/2. After treatment for three weeks, mice were sacrificed and anatomized. The tumour xenografts were collected and weighed. A portion of the tissues were used for protein and total RNA extraction, the others were fixed in 4% paraformaldehyde and then processed for H&E staining or immunohistochemical staining to detect Ki-67 and cleaved-Caspase3.

### Pathological examinations and immunohistochemistry

Pathological examinations were carried out by Servicebio (Wuhan, China). For tissue morphology evaluation, H&E staining was performed on sections from embedded samples. Ki67 and cleaved-Caspase3 expression was assessed at a hot spot under ×200 magnification using Image-Pro Plus software. The number of positive cells per 500 cancer cells was counted in each tumour. Antibodies were as follows: anti-cleaved Caspase3: rabbit monoclonal (Cell Signaling Technology), Ki-67: mouse monoclonal (Dako).

### Statistical analysis

Quantitative RT-PCR and cell apoptosis assays were performed in triplicate. Results were shown as the means ± SE of at least three independent experiments. P-values < 0.05 were considered statistically significant using unpaired, two-tailed Student’s t-tests.

### Ethics approval

All the animal procedures were approved by the Animal Experimentation Ethics Committee of Nanjing University and performed in accordance with institutional policies.

## Electronic supplementary material

Supplementary information

Supplementary dataset S1

Supplementary dataset S2
