# Supplementary material for: Baicalin, the major component of traditional Chinese medicine Scutellaria baicalensis induces colon cancer cell apoptosis through inhibition of oncomiRNAs
Source: Sci Rep. 2018 Sep 27;8:14477. doi: 10.1038/s41598-018-32734-2 (PMC6160418; doi:10.1038/s41598-018-32734-2)

**Baicalin，the major component of traditional Chinese medicine Scutellaria baicalensis induces colon cancer cell apoptosis through inhibition of oncomiRNAs**

Yili Tao^1,*^,Shoubin Zhan^3,*^, Yanbo Wang^3,*^,Geyu Zhou^3^, Hongwei Liang^3^, Xi Chen^3,#^, Hong Shen^2,#^

^1^Nanjing University of Chinese Medicine, 282 HanZhong Road, Nanjing, Jiangsu 210046, China;

^2^Department of Gastroenterology, Affiliated Hospital of Nanjing University of Chinese Medicine, 155 HanZhong Road, Nanjing, Jiangsu 210000, China;

^3^School of Life Sciences, Nanjing University, 163 XianLin Road, Nanjing, Jiangsu 210093, China

^*^these authors contributed equally to this work

^#^ these are corresponding authors

**Supplementary Material**

**Supplementary figure 1: GO: Bioinformatics analysis of target genes and related signalling pathways of the downregulated miRNAs induced by Baicalin. (A)** The target genes of downregulated miRNAs were predicted by miRecords and TarBase, and gene functions were further classified by the GO and DAVID databases based on biological process. **(B-F)** The biological process and pathway enrichment analysis of candidate genes. The top enriched pathways are listed (P value< 0.01).





**Supplementary figure 2: (A)** Flow cytometric analysis of baicalin-induced apop­tosis in SW-480 cells and percentage of apoptotic cells. **(B)** Flow cytometric analysis of baicalin-induced apop­tosis in CACO2 cells and percentage of apoptotic cells. **(C)** The relative change in expression levels of representative miRNAs in the baicalin-treated SW-480 cells compared with controls. **(D)** The relative change in expression levels of representative miRNAs in the baicalin-treated CACO2 cells compared with controls. **(E)** qRT-PCR analysis of downregulated miRNA expression in baicalin treated or untreated SW-480 cells that were transfected with their miRNA-mimics, miRNA-inhibitors, mimic NCs, and inhibitor NCs. **(F)** qRT-PCR analysis of downregulated miRNA expression in baicalin treated or untreated CACO2 cells that were transfected with their miRNA-mimics, miRNA-inhibitors, mimic NCs, and inhibitor NCs. **(G)** Flow cytometric analysis of downregulated miRNA expression in HT-29 cells that were transfected with their miRNA-mimics, miRNA-inhibitors and NCs, and subsequently treated with baicalin. *P<0.05, **P<0.01,***P<0.001.
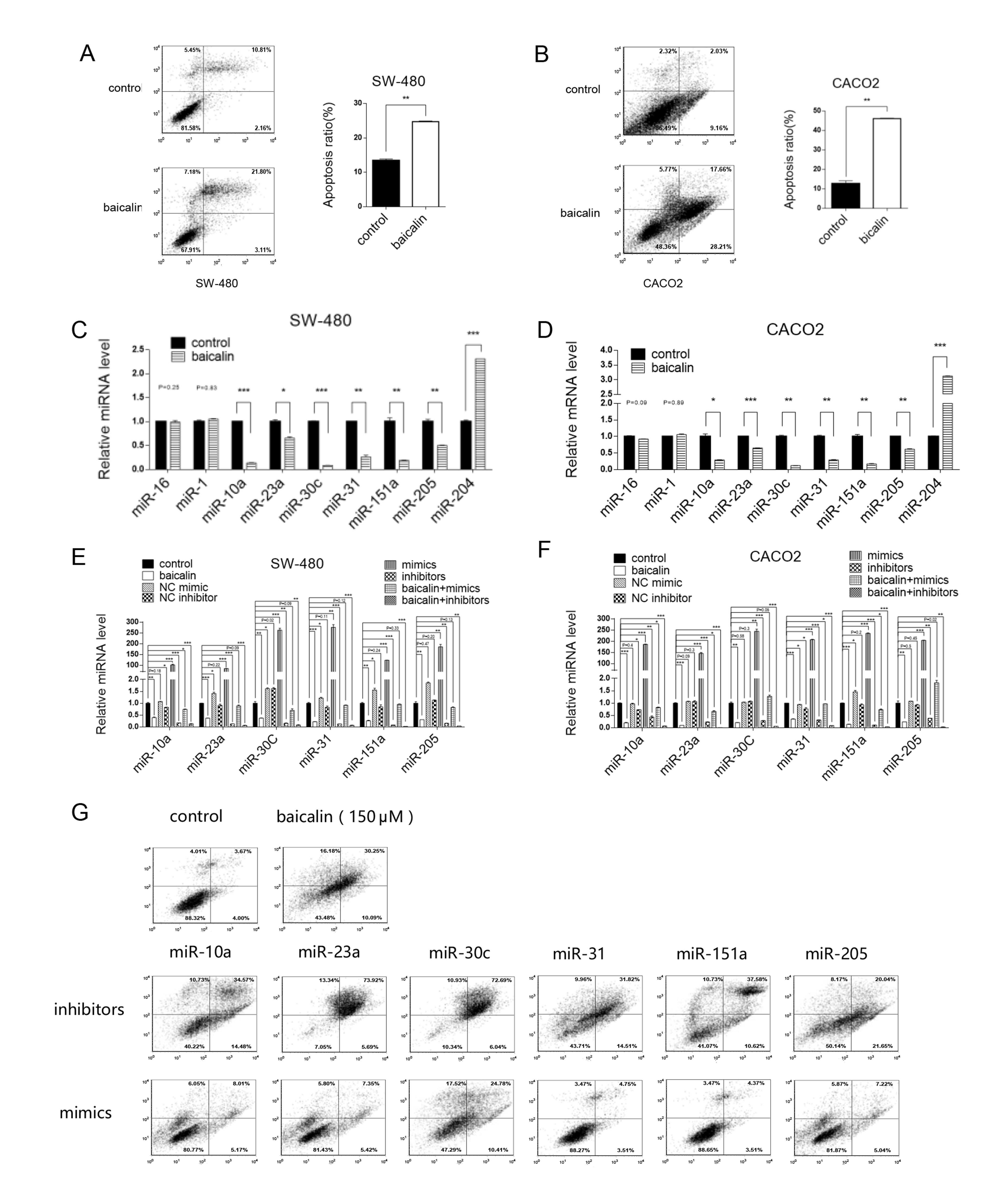


**Supplementary figure 3: (A)** Western blot of c-Myc expression in baicalin-treated SW-480 cells: representative image and quantitative analysis. **(B)** Western blot of c-Myc expression in baicalin-treated CACO2 cells: representative image and quantitative analysis. **(C)** Western blot of c-Myc expression in HT-29 cells transfected with c-Myc siRNAs compared with controls: representative image and quantitative analysis. *P<0.05, **P<0.01,***P<0.001.


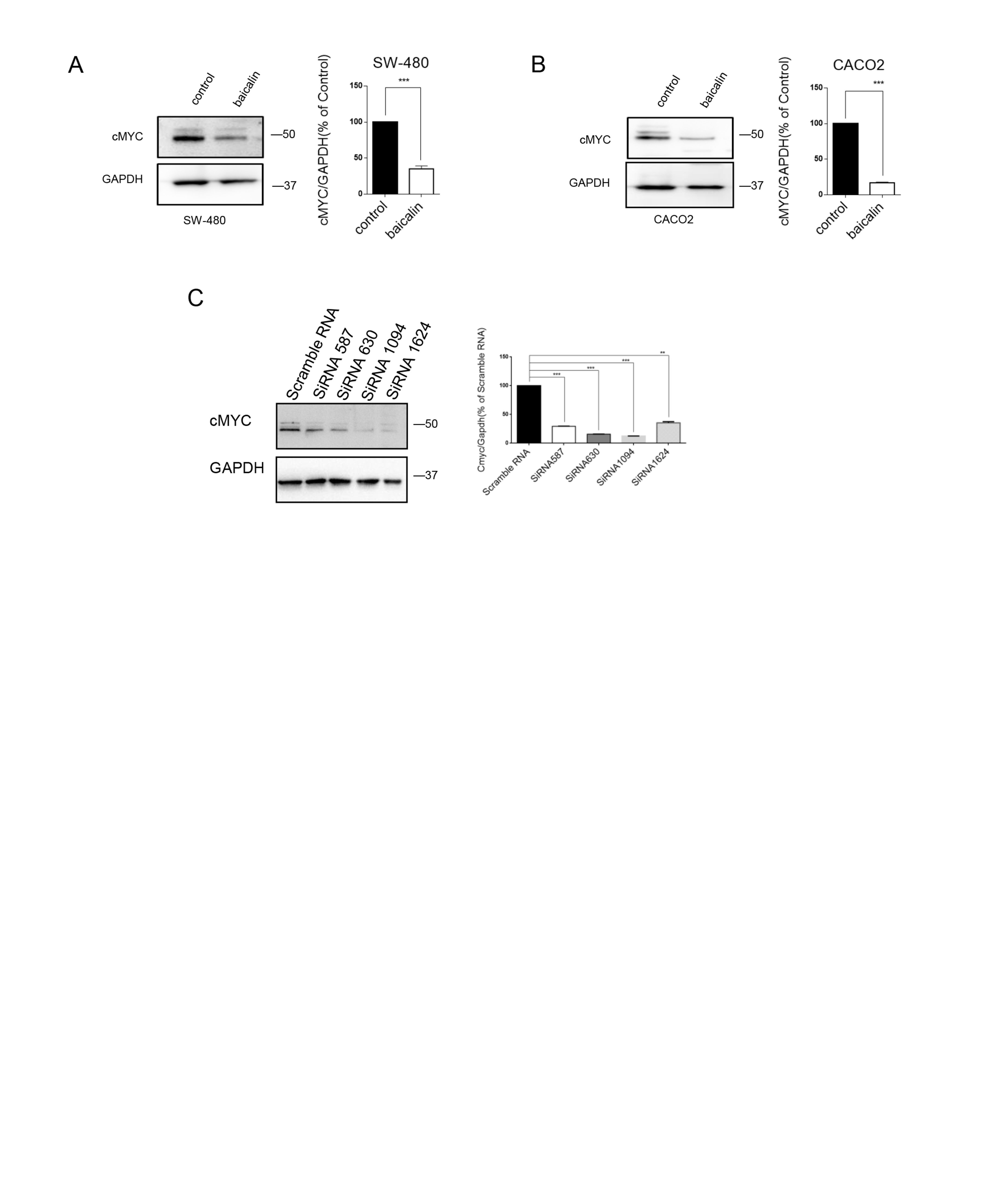

Supplement: Supplementary file 1 — Supplementary information [file 41598_2018_32734_MOESM1_ESM.doc]
